# Major motor and gait deficits with sexual dimorphism in a *Shank3* mutant mouse model

**DOI:** 10.1186/s13229-020-00412-8

**Published:** 2021-01-19

**Authors:** Emmanuel Matas, Alexandre Maisterrena, Mathieu Thabault, Eric Balado, Maureen Francheteau, Anais Balbous, Laurie Galvan, Mohamed Jaber

**Affiliations:** 1 Université de Poitiers, INSERM, Laboratoire de Neurosciences Expérimentales et Cliniques, 86073 Poitiers, France; 2 CHU de Poitiers, 86000 Poitiers, France

**Keywords:** Gait, Sociability, Motor coordination, Cerebellum, Crus I, Crus II, Purkinje cells, mGluR5

## Abstract

**Background:**

Contrasting findings were reported in several animal models with a *Shank3* mutation used to induce various autism spectrum disorder (ASD) symptoms. Here, we aimed at investigating behavioral, cellular, and molecular consequences of a C-terminal (frameshift in exon 21) deletion in *Shank3* protein in mice, a mutation that is also found in clinical conditions and which results in loss of major isoforms of Shank3. A special focus was made on cerebellar related parameters.

**Methods:**

All three genotypes were analyzed [wild type (WT), heterozygote (Shank3+/ΔC) and homozygote (Shank3 ΔC/ΔC)] and males and females were separated into two distinct groups. Motor and social behavior, gait, Purkinje cells (PC) and glutamatergic protein levels were determined. Behavioral and cellular procedures used here were previously validated using two environmental animal models of ASD. ANOVA and post-hoc analysis were used for statistical analysis.

**Results:**

Shank3 ΔC/ΔC mice showed significant impairments in social novelty preference, stereotyped behavior and gait. These were accompanied by a decreased number of PC in restricted cerebellar sub-regions and decreased cerebellar expression of mGluR5. Females Shank3 ΔC/ΔC were less affected by the mutation than males. Shank3+/ΔC mice showed impairments only in social novelty preference, grooming, and decreased mGluR5 expression and that were to a much lesser extent than in Shank3 ΔC/ΔC mice.

**Limitations:**

As *Shank3* mutation is a haploinsufficiency, it is of interest to emphasize that Shank3+/ΔC mice showed only mild to no deficiencies compared to Shank3 ΔC/ΔC.

**Conclusions:**

Our findings indicate that several behavioral, cellular, and molecular parameters are affected in this animal model. The reported deficits are more pronounced in males than in females. Additionally, male Shank3 ΔC/ΔC mice show more pronounced alterations than Shank3+/ΔC. Together with our previous findings in two environmental animal models of ASD, our studies indicate that gait dysfunction constitutes a robust set of motor ASD symptoms that may be considered for implementation in clinical settings as an early and quantitative diagnosis criteria.

## Background

Autism spectrum disorder (ASD) is a set of heterogeneous neurodevelopmental alterations characterized by persistent difficulties in verbal and nonverbal communication with restricted and repetitive patterns of behavior [1]. ASD can be diagnosed during childhood and affects 3 times more males than females [2]. The basis for the male bias in ASD is unknown with theories including the “extreme male brain”, hormonal differences, and genetic influences [3]. For instance, increased levels of testosterone have been correlated with autistic traits in toddlers and young children [4]. Male bias in ASD and intellectual disability have also been associated with rare mutations at chromosome Xp22.11 but that affect less than 1% of patients [5]. In the absence of biological markers, ASD is currently diagnosed based on clinical scales focused on behavioral symptoms. There is presently no cure for ASD but only symptomatic relief to some of its comorbidities such as anxiety, sleep disorders, or seizures [6].

ASD etiology remains elusive, and both genetic and environmental components have been identified [7]. Genetic factors have long been suspected to be involved in ASD, as this is a highly heritable psychiatric disorder [8]. Hundreds of genetic mutations have been associated with ASD in genes such as neurexin (NRXN), neuroligin (NLGN), SH3 and multiple ankyrin repeat domains proteins (SHANK), tuberous sclerosis (TSC)1/2 and fragile X mental retardation (FMR)1, each one contributing to only a small percentage of the disease [9]. Although these mutations only account for approximately 20–30% of ASD cases, many of them seem to converge towards synaptic dysfunction as they are implicated in the formation, the function, and maintenance of synapses, mainly glutamatergic [10, 11]. Based on these findings, the current working hypothesis is that synaptic dysfunction would lead to functional and cognitive impairments observed in ASD [12].

Several genetic mutations linked to these genes have been modeled in mice and were shown to induce behavioral symptoms reminiscent of ASD: deficits in social interaction, impairments in communication, and dysfunctional behaviors [12]. Among these mutations, *Shank3* has received a great deal of attention this last decade [13–15]. Shank (also known as ProSAP) proteins consist of three major isoforms (Shank1, Shank2, and Shank3), and are master scaffolding proteins at excitatory synapses [13, 14]. They interact with more than 30 synaptic proteins through multiple domains and are essential for synaptic formation, glutamate receptor trafficking, and neuronal signaling.

The *Shank3* gene has 22 exons that give rise to numerous mRNA and protein isoforms deriving from multiple intragenic promoters and alternative splicing [11, 13]. Mouse models bearing mutations or deletions in these isoforms have been generated and have been extensively studied this last decade [16]. Although many of these animal models exhibit autism-associated behaviors, such as social deficits and repetitive behaviors, they do so to different extents and with a great deal of variability in terms of neuropathology and behavior [17–27]. These myriad outcomes can be linked to (1) the existence of several *Shank3* animal models in relation with the targeted isoform, (2) lack of systematic use of heterozygote mice, whereas several *Shank3* mutations are in fact a haploinsufficiency in clinical settings, (3) lack of systematic use of female mice in some of these studies and even mixing females with males within the same group of mice, (4) and lack of standardized and robust behavioral procedures.

Here, we aimed at investigating the consequences of a *Shank3* mutation on behavioral parameters that we previously investigated in two different environmental animal models of ASD [28, 29]. These models were obtained following prenatal injection of either valproic acid (Depakine), an anti-epileptic drug targeting the GABA neurotransmission system, or a double stranded RNA (poly IC) mimicking a viral infection and inducing a maternal immune activation. We previously reported that these models recapitulate several ASD symptoms, although at different degrees and magnitude, with a clear sexual dimorphism. Using a full-scale behavioral analysis, we have shown in the VPA model that males and females were differently affected by the treatment with males showing motor, gait and social deficiencies while females showing only motor and gait deficiencies with normal social behavior. At the cellular level, decreased number of cerebellar Purkinje cells (PC) did not affect the same cerebellar sub-regions in males and females. Similar results were obtained with the poly IC injection, although at lower magnitude, where males were also more affected by the treatment than females. These findings recapitulate the spectrum distribution of ASD that affect differently patients in magnitude and in symptomatology with a clear sexual dimorphism.

In line, we investigate here behavioral, cellular, and molecular consequences of lack of *Shank3* expression [17]. In this animal model Shank3 is lacking the entire C-terminal region, including the Homer-binding site in the sterile alpha motif (SAM) domain. This results in a partial or total loss of the major naturally occurring isoforms of Shank3 proteins in heterozygotes and homozygotes, respectively. This mutation was chosen as it has strong construct validity given that it mimics a human mutation, which is not the case of several other *Shank3* mutations in mice. Major behavioral deficits were reported in these mice, including social preference deficits and repetitive behaviors, but not always using both males and females or Shank3+/ΔC and Shank3 ΔC/ΔC mice [22–25]. Here we used wild type, Shank3+/ΔC, and Shank3 ΔC/ΔC mice, and we analyzed males and females separately. A special focus was made on cerebellar-related parameters as we have previously shown that motor dysfunction and gait deficits are strongly correlated with the degree of severity of ASD in terms of social symptoms and loss of PC. Standardized behavioral procedures were used and were previously validated in two environmental models of ASD using a prenatal injection of either valproic acid (VPA) or the double-stranded RNA Poly: IC, inducing a maternal immune activation (MIA) [28, 29].

We report here that loss of major Shank3 isoforms induces several social, motor, and gait behavioral phenotypes reminiscent of ASD symptoms in humans. These are accompanied by a restricted decrease in both cerebellar PC cell number and mGluR5 levels. While social interaction deficits were observed in Shank3 ΔC/ΔC and Shank3+/ΔC, in both males and females, other parameters investigated affected mostly male Shank3 ΔC/ΔC mice.

## Methods

### Design, and setting

Shank3+/ΔC mice with a C-terminal deletion of *Shank3* at exon 21 (Shank3tm1.1Pfw/J) were obtained from Jackson laboratories and are on a C57BL/6J genetic background (Jackson Laboratories, USA); they were originally donated by Paul Worley from Johns Hopkins University School of Medicine [17]. Shank3+/ΔC mice were mated to generate all mice of all three genotypes: WT, Shank3+/ΔC, and Shank3 ΔC/ΔC. Sex and age-matched pups were separated from the dams on postnatal day 28 (P28) and were then genotyped and raised by groups of 4 of the same genotype in a randomized fashion to avoid litter effects. All litters of the three genotypes and two sexes were used for this study. Outliers and mice that did not perform a given test appropriately (not finishing the walking beam, jumping out of the apparatus, stalling or reversing during gait…) were taken out from the study. Comprehensive behavioral screening was performed between postnatal days 50 and 60 (P50 and P60) as we previously described in two environmental ASD mouse models [28, 29]. Mice were tested during their light cycle and the investigators were blind to genotype. At the end of the behavioral experiments, mice were sacrificed and brains were harvested for histological analysis, RT-PCRq, or western blotting experiments.

### Animals

Animal housing and experimental procedures were performed under the European Union directive (2010/63/EU) and validated by the regional ethical committee (Approval # 2015020415093780). Mice were housed in ventilated cages with access to food and water ad libitum. Room temperature was maintained at 23 °C on a 12 h light/dark cycle. Offspring were segregated into 6 different groups: wild type males (*n* = 47), Shank3+/ΔC males (*n* = 71), Shank3 ΔC/ΔC males (*n* = 27), wild type females (*n* = 39), Shank3+/ΔC females (*n* = 58) and Shank3 ΔC/ΔC females (*n* = 26). A set of these mice was used for every procedure, as detailed below.

### Behavioral analysis

All behavioral tests were performed as we previously described in detail in two different environmental ASD mice models [28, 29].

*Social interaction* was assessed using the three-chambers test (3-CT) in an apparatus that consists of a Plexiglass box (60 × 45 × 22 cm) partitioned into three chambers with retractable doorways. Mice were monitored using an unbiased automated video tracking system (VideoTrack, Viewpoint-France) which calculates several parameters for each observed animal such as position, speed, trajectory, and various other behaviors set up by the experimenter. Data were retrieved using the VideoTrack 3.2 software (Viewpoint-France). Mice were habituated to the 3-CT for 20 min 2 days before phase 1 that consists of two identical non-social stimuli (inverted wire-cups) placed in the opposite chambers. The second phase comprises a non-social stimulus and a social stimulus (a naïve mouse with no previous contact with the tested mouse). The naïve mouse is of the same sex and age and is usually used for two tests per day at the most, with a 2–3 h rest between tests; no mouse is used more than 2 consecutive days. In the third phase, an additional and novel mouse was placed in the cup present in the opposite chamber. After a habituation phase of 10 min in the central chamber, mice underwent the three phases of the experiment, with each phase being of 10 min each during which time spent in each chamber and around each cup was recorded. Mice used for this procedure were Wild type males (*n* = 13), Shank3+/ΔC males (*n* = 20), Shank3 ΔC/ΔC males (*n* = 18), wild type females (*n* = 14), Shank3+/ΔC females (*n* = 12), Shank3 ΔC/ΔC females (*n* = 16).

*Spontaneous grooming behavior* was scored in mice placed individually in a clean and transparent cylinder with house bedding. Each mouse was videotaped and rated for 10 min. Cumulative time spent grooming and grooming frequency were evaluated. Wild type males (*n* = 20), Shank3+/ΔC males (*n* = 12), Shank3 ΔC/ΔC males (*n* = 7), wild type females (*n* = 12), Shank3+/ΔC females (*n* = 12), Shank3 ΔC/ΔC females (*n* = 13).

*Motor coordination* was evaluated using the challenging beam test and which consists of four Plexiglass sections (25 cm each) starting with a width of 3.5 cm and gradually narrowed to 0.5 by 1 cm decrements. Mice were first trained for 2 days to traverse the beam, starting at the widest section and ending at the narrowest section that led into the home cage. On the test day, a mesh grid (1 cm squares) was placed over the beam surface. Mice were videotaped while traversing the grid-surfaced beam for five trials. Mice that stalled during the beam walk or tried to reverse course were not included in the final analysis. Time to traverse, errors, number of steps, and errors per step made by each mouse were measured and averaged. Wild type males (*n* = 10), Shank3+/ΔC males (*n* = 20), Shank3 ΔC/ΔC males (*n* = 14), wild type females (*n* = 14), Shank3+/ΔC females (*n* = 17), Shank3 ΔC/ΔC females (*n* = 12).

*Gait* was analyzed during spontaneous walk using an automated gait analysis system (Gaitlab, Viewpoint, France). The apparatus is made of a 1.5 m long glass corridor with dim green light beamed into the glass walkway. Each mouse was assessed individually for 3 consecutive runs. Mice that stalled during the beam walk or tried to reverse course were not included in the final analysis. All gait parameters were analyzed with a special focus on (1) stride length: distance between two consecutive placements of the same paw, (2) limb base of support: distance between two pair prints at contact during each step cycle, and (3) pair gap: gap between the placement of the two trailing feet, which measures spatial coordination between the two pairs. Wild type males (*n* = 11), Shank3+/ΔC males (*n* = 27), Shank3 ΔC/ΔC males (*n* = 15), wild type females (*n* = 11), Shank3+/ΔC females (*n* = 21), Shank3 ΔC/ΔC females (*n* = 12).

### Tissue processing and immunohistochemistry

Males and females from each genotype were randomly selected for either histological, mRNA, or protein analysis. For histological analysis, mice (wild type males (*n* = 8), Shank3+/ΔC males (*n* = 11), Shank3 ΔC/ΔC males (*n* = 8), wild type females (*n* = 8), Shank3+/ΔC females (*n* = 9), Shank3 ΔC/ΔC females (*n* = 6)) were deeply anesthetized with ketamine-xylazine (120–20 mg/kg) and transcardially perfused with 0.9% saline at 37 °C followed by 4% paraformaldehyde (PFA) at 4 °C. Brains were post-fixed in 4% PFA at 4 °C for 24 h before cryoprotection in 30% sucrose for 48 h. Serial 50 µm (cerebellum) free-floating sections were collected and stored at − 20 °C until use in an anti-freeze solution. Every fourth cerebellar section was mounted on gelatin-coated slides for PC quantification. PC were identified based on their morphology on cresyl violet staining. The PC phenotype was confirmed using calbindin immunohistochemistry (1:2500; Swant, Cb-38a) [30]. Stereological estimates were performed using the optical fractionator method and systematic random sampling to obtain the total number of cerebellar PC. Each region of interest was outlined based on the Franklin and Paxinos’s mouse brain atlas [31] at 2.5 × objective. Neurons were counted at 40 × objective using the Mercator image analysis system (Explora Nova, France). Upper and lower guard zones of 1 µm were set at the top and bottom of the section to exclude lost profiles and each neuron or visible nucleus was counted as previously described [28, 29].

### Quantitative immunoblot analysis

Mice from each group [wild type males (*n* = 4), Shank3+/ΔC males (*n* = 8), Shank3 ΔC/ΔC males (*n* = 8), wild type females (*n* = 5), Shank3+/ΔC females (*n* = 8), Shank3 ΔC/ΔC females (*n* = 9)] were sacrificed, brains were retrieved, and cerebellum region was dissected out and frozen in – 80 C until use. For control purposes, we also used cerebella obtained from offspring of pregnant mice that received VPA injections at E12.5, as previously described [28]. Proteins were extracted using a 1% sodium dodecyl sulfate (SDS) solution in Tris Hcl 0.1 M with EDTA 0.01 M and PMSF, protease inhibitor, and phosphatase inhibitor at 1%. Equal amounts of proteins from cerebellum lysates were separated by SDS-PAGE and migrated proteins were transferred to nitrocellulose membranes (Bio-Rad). After blocking the membranes at room temperature for 1 h in Tris Buffer Solution with Tween-20 0,1 M (TBST) and (pH 7.4, TBS) 5% non-fat milk, the blots were incubated with corresponding primary antibodies at room temperature for 3 h. The blots were then washed three times in TBST and incubated with HRP-conjugated secondary antibodies overnight at 4 °C. Following 3 washes in TBST, the blots were incubated with ECL reagent (GE Healthcare Life Sciences, NJ, USA). For quantification, the films were scanned by a PXi image system and gray signals were analyzed by GeneTools software (Syngene, Cambridge, UK), and normalized to that of corresponding internal controls (actin or β-tubulin III). The primary antibodies mTOR 7C10, P-mTOR ser 2448, ERK1/2, and P-ERK1/2 (Cell Signaling Technology, Leiden, The Netherlands) were used at the dilution 1/1000 to 1/1500. NR2A, NR2B, GluR1, and GAPDH antibodies (Millipore, Paris, France) were used at the dilution of 1/500 to 1/1500. The following dilutions were used for antibodies against CAMKII (1/1000), α-tubulin (1/10,000), and β-actin (1/10,000) (Sigma-Aldrich, Lyon, France). Shank3 and mGluR5 antibodies (Abcam, Paris, France) were used at the dilution of 1/500 and 1/5000, respectively. NR1 antibodies (Antibodies Incorporated, California, USA) were used at the dilution of 1/500. GluR2 antibodies (Neuromab, California, USA) were used at the dilution of 1/1000.

### RT-PCRq analysis of mGluR5 mRNA levels

Mice from each group (wild type males (*n* = 7), Shank3+/ΔC males (*n* = 8), Shank3 ΔC/ΔC males (*n* = 8), wild type females (*n* = 7), Shank3+/ΔC females (*n* = 8), Shank3 ΔC/ΔC females (*n* = 8)) were sacrificed, brains were retrieved, and cerebellum region was dissected out and frozen in -80C until use. Total RNA was isolated using TRIzol Reagent/chloroform, then purified using a NucleoSpin RNA kit (Macherey-Nagel) and quantified using NanoDrop ND-1000 spectrophotometer (Thermo Fisher Scientific, USA). Reverse transcription was performed on 1 μg of total RNA for each sample using Verso cDNA Synthesis kit (Thermo Fischer Scientific). qPCR was performed on LightCycler 480 system (Roche Diagnostics, France). Ct values were averaged from triplicates. Results were subtracted to the mean Ct of the housekeeping genes Gapdh, Beta actin and cyclophilin A (ΔCt) and log transformed (2^−ΔCt^). mGluR5 primers sequence was designed using Primer-Blast (NCBI); primers were synthesized by IDT: 5′-aggacagataaaggtgatcc-3′ and5′-agatactggactgggatcaa-3′.

### Statistical analyses

Data are expressed as mean ± Standard Error of the Mean (SEM) and analyzed using GraphPad Prism-7 software (La Jolla, California, USA). Data followed normal distribution as evidenced by the Shapiro–Wilks normality test with the hypothesis for normality rejected at a *p* value less or equal to 0.05. Data were analyzed using Student’s *t* test, one-way or two-way analysis of variance (ANOVA) when relevant and as stated for each experiment. Tukey’s or Fisher’s LSD post-hoc multiple comparisons were applied. Outliers were identified using the Grubbs statistical test with alpha at 0.05. For all analyses, a *p* value < 0.05 was considered significant.

## Results

### Shank3+/ΔC and Shank3 ΔC /ΔC mice show impairments in social novelty preference

**Fig. 1:**
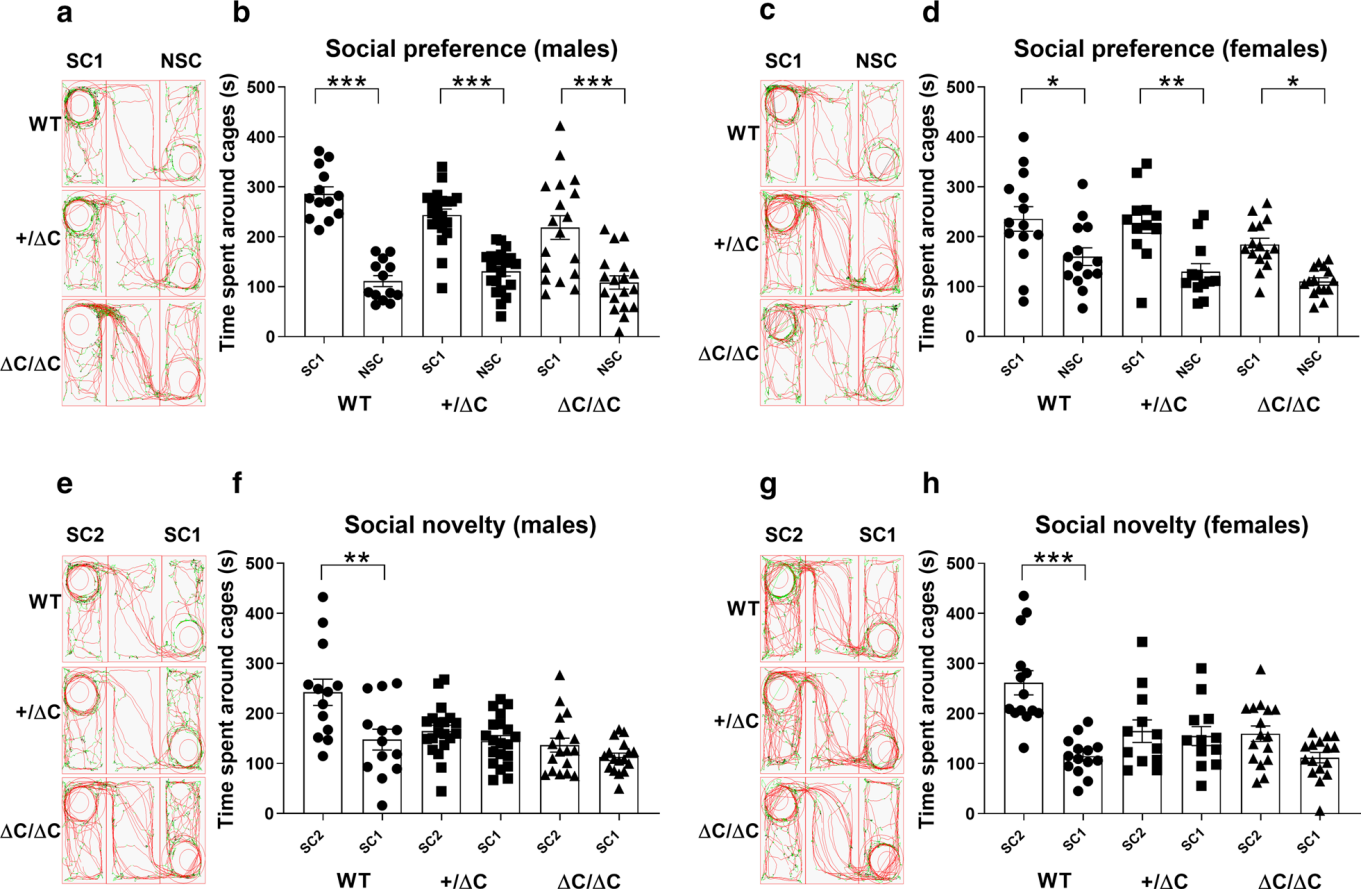
Social preference in *Shank3* mice assessed through the three-chambers test (3-CT). **a**–**c**–**e**–**g** Representative tracks of mice in the 3-CT. **b**–**d**–**f**–**h** Social behavior analysis. **b**–**d**. Following habituation, mice were put in the 3-CT with one chamber containing an encaged mouse (social chamber 1: SC1) and one chamber with an empty cage (Non-social chamber: NSC) to evaluate social preference. Note that all mice, whatever the sex and the genotype, spent more time in SC1 than in NSC. **f**–**h** Then, a novel mouse was introduced in the chamber 2 (social chamber 2: SC2) in addition to the encaged mouse in SC1 to evaluate social novelty recognition. Note that Shank3 ΔC/ΔC and Shank3+/ΔC mice of both sexes spend equal time interacting with the novel mouse in SC2 than with the mouse in SC1. In comparison, wild type mice spent more time interacting with SC2 than with SC1. All data are expressed as means ± SEM; One-way ANOVA followed by Tukey’s multiple analysis was performed (*p < 0.05, **p < 0.01, ***p < 0.001). WT males *n* = 13; Shank3+/ΔC males *n* = 20; Shank3 ΔC/ΔC males, *n* = 18; WT females *n* = 14; Shank3+/ΔC females n = 12; Shank3 ΔC/ΔC females, *n* = 16

Social preference and motivation in mice have strong face validity to simple social approach behaviors in humans, which are frequently impaired in ASD [32]. We have used here a standardized 3-CT procedure, as previously described [28, 29], and evaluated social preference and novelty. Surprisingly, we observed social preferences with all genotypes (Males: One-way ANOVA, [F(5,96) = 23.69, *p* < 0.0001]; Females: One-way ANOVA, [F(5,76) = 8.852, *p* < 0.0001]) and in both males (Tukey’s post-hoc test: WT, *p* < 0.0001; Shank3+/ΔC, *p* < 0.0001; Shank3 ΔC/ΔC, *p* < 0.0001) and females (Tukey’s post-hoc test: WT, *p* = 0.026; Shank3+/ΔC, *p* = 0.0039; Shank3 ΔC/ΔC, *p* = 0.023) (Fig. 1b, d). Indeed, mice from all genotypes and sex spent more time around the cage that contained another mouse than around an empty cage (phase 2). Preference for social novelty is defined as a significant propensity to spend more time with a new mouse than with a familiar mouse. When a novel mouse was then introduced in the opposite chamber (phase 3), only WT mice of both sexes showed social preference to the novel mouse (Males: One-way ANOVA, [F(5,94) = 7.729, *p* < 0.0001]; Females: One-way ANOVA, [F(5,78) = 10.21, *p* < 0.0001]). Tukey’s post-hoc test revealed a significant increase (males: + 94.65%; females: + 146.70%) in the time spent within the cage area of the novel mouse only in the WT group of males (*p* = 0.0016) and females (*p* < 0.0001) but not in either the Shank3+/ΔC or the ΔC/ΔC groups of males or females (Fig. 1f, h).

These results indicate that Shank3+/ΔC and Shank3 ΔC/ΔC males and females show social preference and motivation but have impaired preference for social novelty as they show no preference for a newly introduced mouse compared to a mouse with which they have previously interacted.

### Shank3 ΔC/ΔC mice show increased self-grooming frequency

**Fig. 2:**
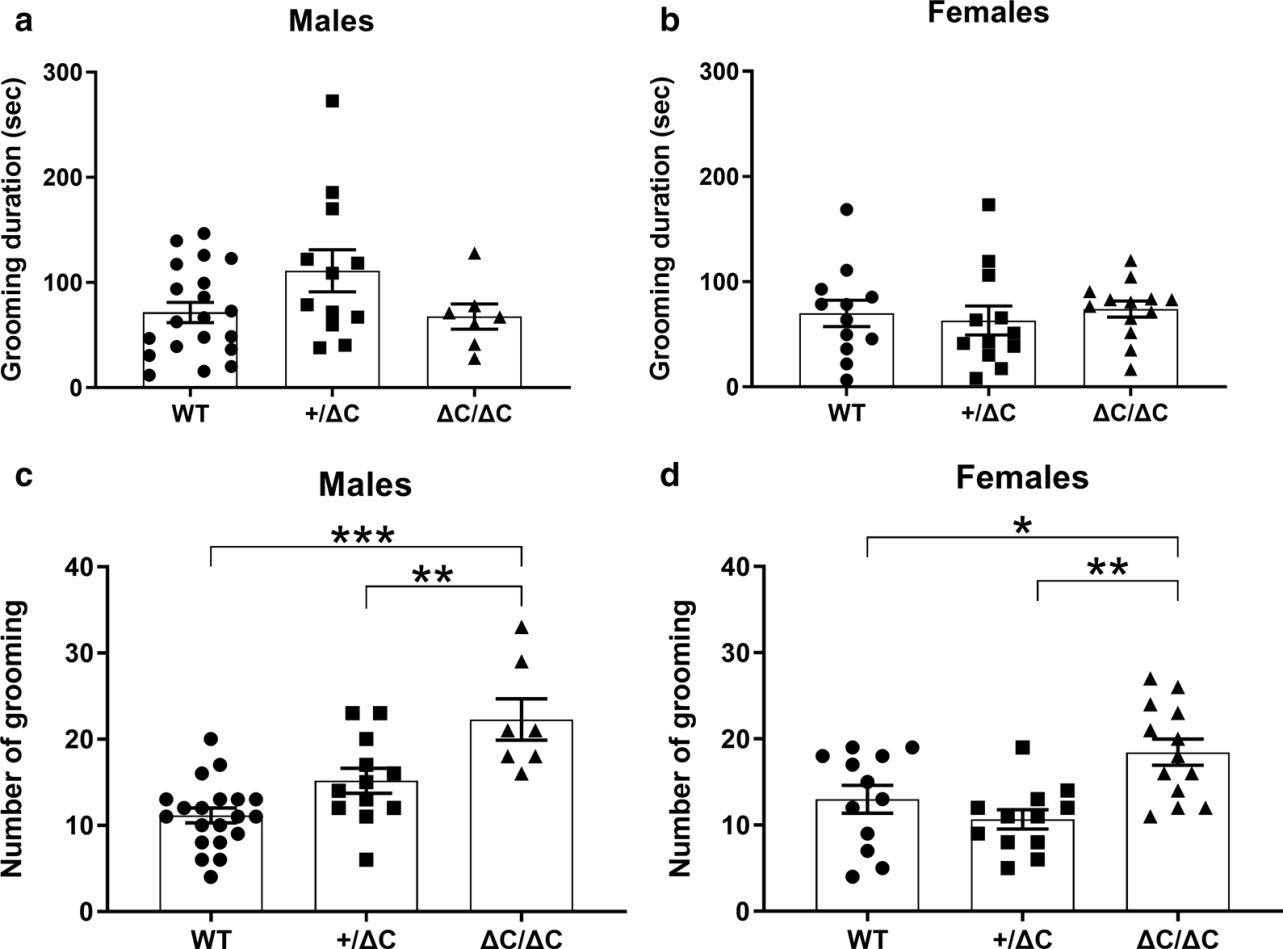
Stereotyped behavior in *Shank3* mice. **a**–**d** Grooming time and frequency in *Shank3* mice. Mice were placed in a clean cylinder and videotaped. **a**–**c** There was no difference in the time spent grooming within all groups of mice. **b**–**d** However, compared to WT, grooming frequency was increased in Shank3 ΔC/ΔC and Shank3+/ΔC males. Only Shank3 ΔC/ΔC females showed increased grooming compared to WT and Shank3+/ΔC (B). All data are expressed as means ± SEM. One-way ANOVA followed by Tukey’s multiple analysis was performed (*p < 0.05, **p < 0.01, ***p < 0.001) WT males *n* = 20; Shank3+/ΔC males *n* = 12; Shank3 ΔC/ΔC males, *n* = 7; WT females *n* = 12; Shank3+/ΔC females *n* = 12; Shank3 ΔC/ΔC females, *n* = 13

Self-grooming is an innate behavior in rodents and its alteration is used as an indication of compulsive and repetitive behavior [33]. Motor stereotypies, repetitive and uncontrollable motor behaviors are among the core symptoms of ASD. Most *Shank3* mouse models were shown to display increased self-grooming although with conflicting results in relation to the genotype and the sex of mice [17, 18, 21–24]. Here we have assessed grooming in a novel environment (a transparent cylinder) with home cage bedding (Fig. 2). We report that time spent grooming is not significantly different in relation with genotype in males (One-way ANOVA, [F(2,36) = 2.653, *p* = 0.0842]) and females (One-way ANOVA, [F(2,34) = 0.232, *p* = 0.79]) (Fig. 2a, b). However, there was a significant effect in the number of grooming episodes in relation with genotype (Males: One-way ANOVA, [F(2,36) = 14.71, *p* < 0.0001]; Females: One-way ANOVA, [F(2,34) = 7.951, *p* = 0.001]). Indeed, Tukey’s post-hoc analysis showed that the number of grooming episodes increased in both the Shank3 ΔC/ΔC groups of males and female mice (*p* < 0.0001 and *p* = 0.03, respectively) (Fig. 2c, d). These results are in line with previous reports in several mouse models bearing a *Shank3* mutation indicating increased grooming in these mouse models as a key behavioral feature.

### Shank3 ΔC/ΔC mice show motor coordination deficits

**Fig. 3:**
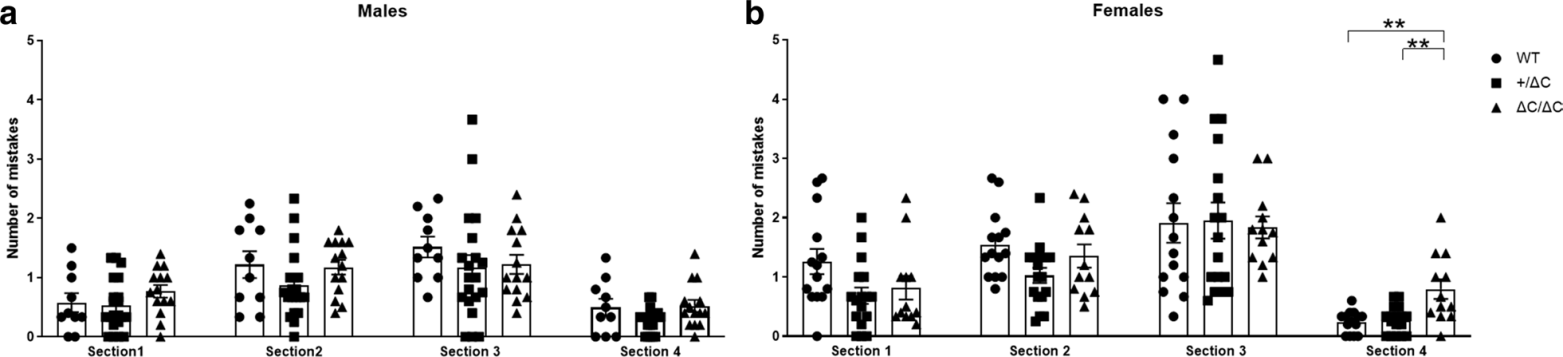
Motor coordination deficits in *Shank3* mice. Time to traverse the beam was only slightly affected in the last section of the beam, and which is the narrower, in Shank3 ΔC/ΔC females. This is indicative of motor coordination deficits. All data are expressed as means ± SEM. One-way ANOVA followed by Tukey’s multiple analysis was performed (*p < 0.05, **p < 0.01, ***p < 0.001) WT males *n* = 10; Shank3+/ΔC males *n* = 20; Shank3 ΔC/ΔC males, *n* = 14; WT females *n* = 14; Shank3+/ΔC females *n* = 17; Shank3 ΔC/ΔC females, *n* = 12

Motor coordination deficits were found in ASD patients but are not used in the diagnosis criteria [34]. Motor coordination can be explored using the challenging beam test, usually performed in animal models of pathologies affecting motor behavior such as Parkinson’s disease [35]. We have recently implemented and validated this procedure in two environmental mouse models of ASD [28, 29]. Here, we report that Shank3 ΔC/ΔC mice display mild motor coordination deficits (Fig. 3). Indeed, there was a significant effect of genotype (One-way ANOVA, [F(2,40) = 10.07, *p* = 0.001]) in the number of mistakes while traversing the last, and thus the narrowest, section of the beam in the Shank3 ΔC/ΔC female group (Tukey’s post-hoc analysis, *p* = 0.0008) (Fig. 3b). In male Shank3 ΔC/ΔC mice, there was clearly a tendency towards motor deficits but that did not reach significance (One-way ANOVA, [F(2,41) = 2.758, *p* = 0.075) (Fig. 3a). Shank3+/ΔC mice did not show any deficits, whatever the sex and the section of the beam.

### Shank3 ΔC/ΔC mice show gait abnormalities

**Fig. 4:**
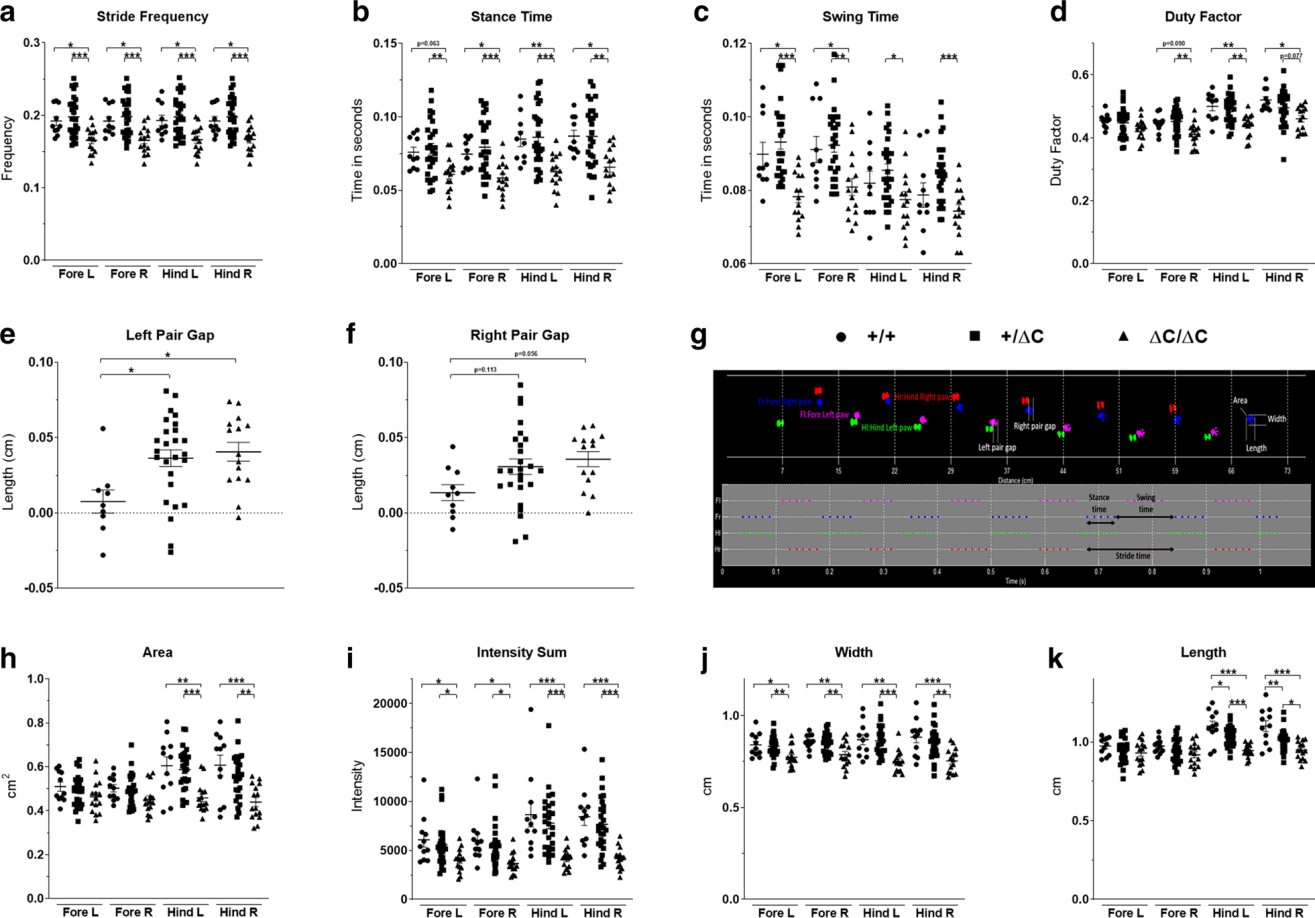
Gait analysis in *Shank3* males. Gait was analyzed during spontaneous walk using an automated gait analysis system (Viewpoint, France). Ten parameters were analyzed: **a** stride frequency, **b** stance time, **c** swing time, **d** duty factor, **e**, **f** pair gap, **h** area, **i** intensity sum, **j** width, **k** length and **g** representative spatio-temporal gait analysis. All data are expressed as means ± SEM. One way ANOVA followed by Tukey’s multiple analysis was performed. (*p < 0.05, **p < 0.01, ***p < 0.001) WT males *n* = 11; Shank3+/ΔC males *n* = 27; Shank3 ΔC/ΔC males, *n* = 15

**Fig. 5:**
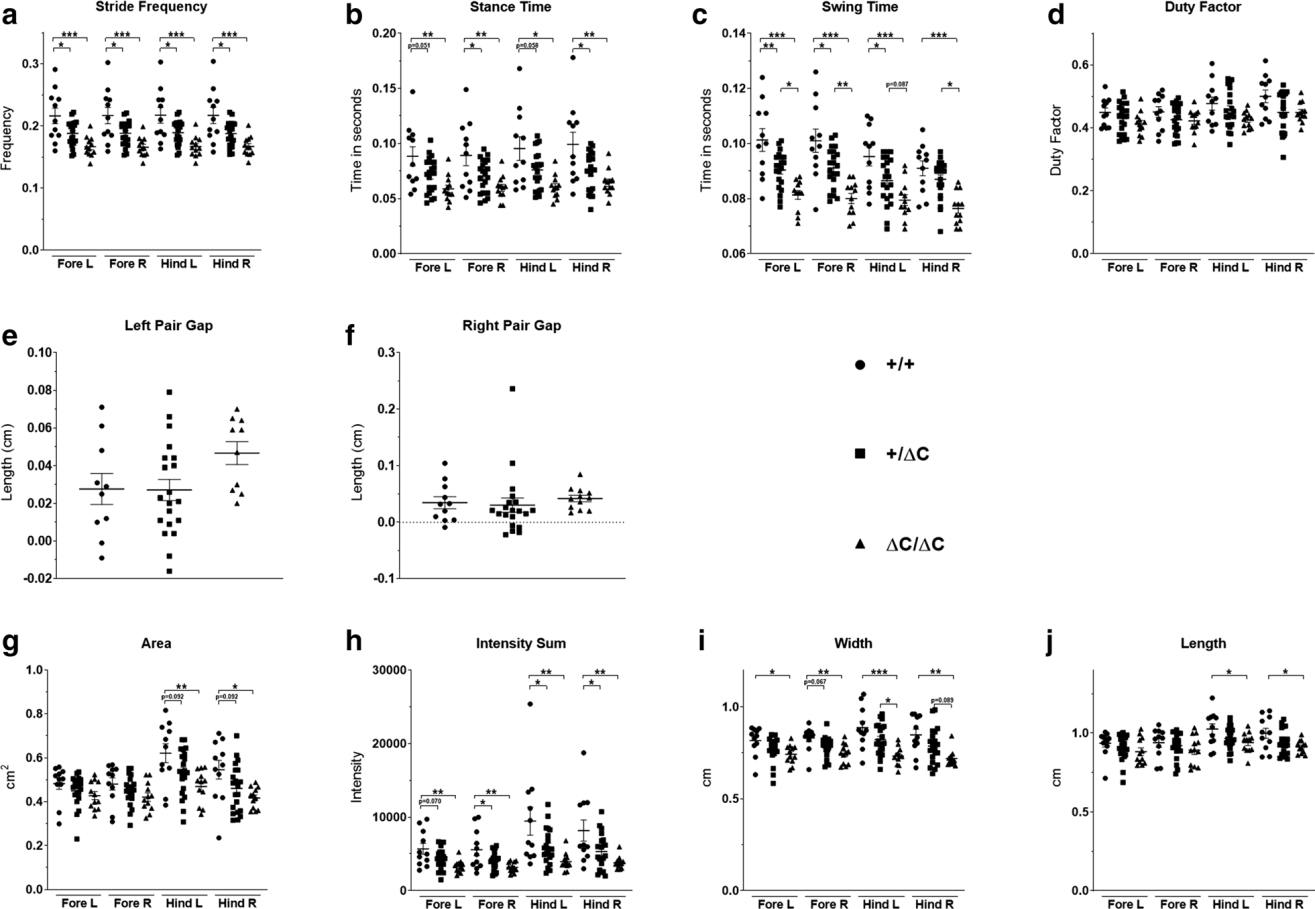
Gait analysis in *Shank3* females. Gait was analyzed during spontaneous walk as with males. The following parameters were analyzed: **a** stride frequency, **b** stance time, **c** swing time, **d** duty factor, **e**, **f** pair gap, **g** area, **h** intensity sum, **i** width, **j** length. All data are expressed as means ± SEM. One way ANOVA followed by Tukey’s multiple analysis was performed. (*p < 0.05, **p < 0.01, ***p < 0.001) WT females *n* = 11; Shank3+/ΔC females *n* = 21; Shank3 ΔC/ΔC females, *n* = 12

Although gait disturbances and motor development delays have been constantly reported in ASD patients [36], they are still not included in the diagnosis criteria. We have previously shown in the VPA and Poly IC ASD mouse models significant alterations in several gait parameters explored here (Figs. 4, 5).

We found that Shank3 ΔC/ΔC mice display several gait abnormalities in greater number in males than in females. For example, in the male group these parameters include the following: (1) swing time (fore left paw: One-way ANOVA, [F(2,49) = 12.48, *p* < 0.0001] and fore right paw: One-way ANOVA, [F(2,49) = 6.869, *p* = 0.002]). Tukey’s multiple analysis showed that fore left paw was significantly decreased by 12.84% in Shank3 ΔC/ΔC mice compared to wild type (*p* = 0.011) and the fore right paw was significantly decreased by 11.23% (*p* = 0.036) (Fig. 4c); (2) stance time was significantly affected in Shank3 ΔC/ΔC mice for the fore right, hind left and hind right paws whereas only a trend towards a decrease was observed for the fore left paw as revealed by post-hoc analysis. Tukey’s multiple analysis showed a significant decrease of the stance time by 26.18% for Shank3 ΔC/ΔC mice hind left paw [F(2,49) = 9.003, *p* > 0.01] (Fig. 4b); (3) stride frequency was also affected (One-way ANOVA followed by Tukey’s multiple analysis (WT vs Shank3 ΔC/ΔC); Fore left paw: [F(2,49) = 9.431, *p* = 0.02], fore right paw: [F(2,49) = 9.253, *p* = 0.02], hind left paw: [F(2,49) = 8.265, *p* = 0.03] and hind right paw: [F(2,49) = 8.675, *p* = 0.03]) (Fig. 4a); (4) left pair gap (One-way ANOVA followed by Tukey’s multiple analysis (WT vs Shank3 ΔC/ΔC): ([F(2,47) = 5.026, *p* = 0.012]) (Fig. 4e, f) and also area (Fig. 4h), width (Fig. 4j) and length (Fig. 4k) of the footsteps. Duty factor, which is the ratio of the limb stance time and limb stride time, was decreased only in Shank3 ΔC/ΔC males (One-way ANOVA followed by Tukey’s multiple analysis when applicable: Fore left paw: [F(2,49) = 1.62, *p* = 0.21]. Fore right paw: [F(2,49) = 5.372, *p* = 0.1], hind left paw: [F(2,49) = 8.365, *p* = 0.01] and hind right paw: [F(2,49) = 4.894, *p* = 0.011]. Intensity sum, defined as the sum of the work exerted through body movement and the resting, was decreased in both male and female Shank3 ΔC/ΔC compared to wild type (One-way ANOVA followed by Tukey’s multiple analysis: ([F(2,50) = 4.423, *p* = 0.027] and [F(2,41) = 6.165, *p* = 0.003], respectively) (Figs. 4i, 5h).

Similar alterations were also observed in the Shank3 ΔC/ΔC female group but implicating less gait parameters than in males. For instance, Tukey’s multiple analysis showed a significant decrease of the swing time by 19.71% for Shank3 ΔC/ΔC mice fore left paw [F(2,41) = 15.25, *p* < 0.0001] and by for 20.79% the fore left paw [F(2,41) = 15.01, *p* < 0.0001]. In addition, one-way ANOVA followed by Tukey’s multiple analysis showed that swing time fore left paw [F(2,41) = 15.25, *p* = 0.004] and swing time fore right paw [F(2,41) = 15.01, *p* = 0.016] were significantly reduced in female Shank3+/ΔC mice compared to WT (Fig. 5c). Stance time was also significantly affected in Shank3 ΔC/ΔC and Shank3+/ΔC females. For example, One-way ANOVA followed by Tukey’s multiple analysis showed the following differences between WT and Shank3 ΔC/ΔC in fore left paw [F(2,41) = 6.908, *p* = 0.002], fore right paw [F(2,41) = 6.797, *p* = 0.002], fore left paw [F(2,41) = 6.908, *p* = 0.05] and fore right paw [F(2,41) = 6.8, *p* < 0.05]) (Fig. 5b). For stride frequency, a significant decrease was observed for the fore and hind paws of Shank3 ΔC/ΔC females compared to WT: fore left paw [F(2,41) = 10.29, *p* = 0.0001], fore right paw [F(2,41) = 10.35, *p* = 0.0001], hind left paw [F(2,41) = 10.55, *p* = 0.0001] and hind right paw [F(2,41) = 10.09, *p* = 0.001]). There were also differences in this parameter in Shank3+/ΔC females compared to WT: Fore left paw [F(2,41) = 10.29, *p* = 0.017], fore right paw [F(2,41) = 10.35, *p* = 0.018], hind left paw [F(2,41) = 10.55, *p* = 0.018] and hind right paw [F(2,41) = 10.09, *p* = 0.015]) (Fig. 5a). Duty factor was not affected in Shank3 ΔC/ΔC females compared to WT: Fore left paw [F(2,41) = 1.861, *p* = 0.17], fore right paw [F(2,41) = 1.277, *p* = 0.29], hind left paw [F(2,41) = 2.524, *p* = 0.09] and hind right paw [F(2,41) = 3.206, *p* = 0.051]) (Fig. 5d).

Taken together, gait analysis indicates major abnormalities mainly in Shank3 ΔC/ΔC mice, with more parameters encountered in males than in females. These abnormalities are often of spatio-temporal nature rather than anatomical [37].

### Male Shank3 ΔC/ΔC mice show decreased Purkinje cell number

We aimed at determining the effect of *Shank3* mutation on cell numbers in the Crus I and Crus II cerebellar sub-regions that are known to play a major role in movement regulation, exploratory behavior, stereotyped and repetitive behaviors [38] but also in social interactions [39]. Several clinical studies in ASD [40, 41] and explorations in animal models, including our own [28, 29, 42], have consistently shown a decrease in the number of these neurons in the cerebellum and mainly within the Crus I and/or Crus II sub-regions.

**Fig. 6:**
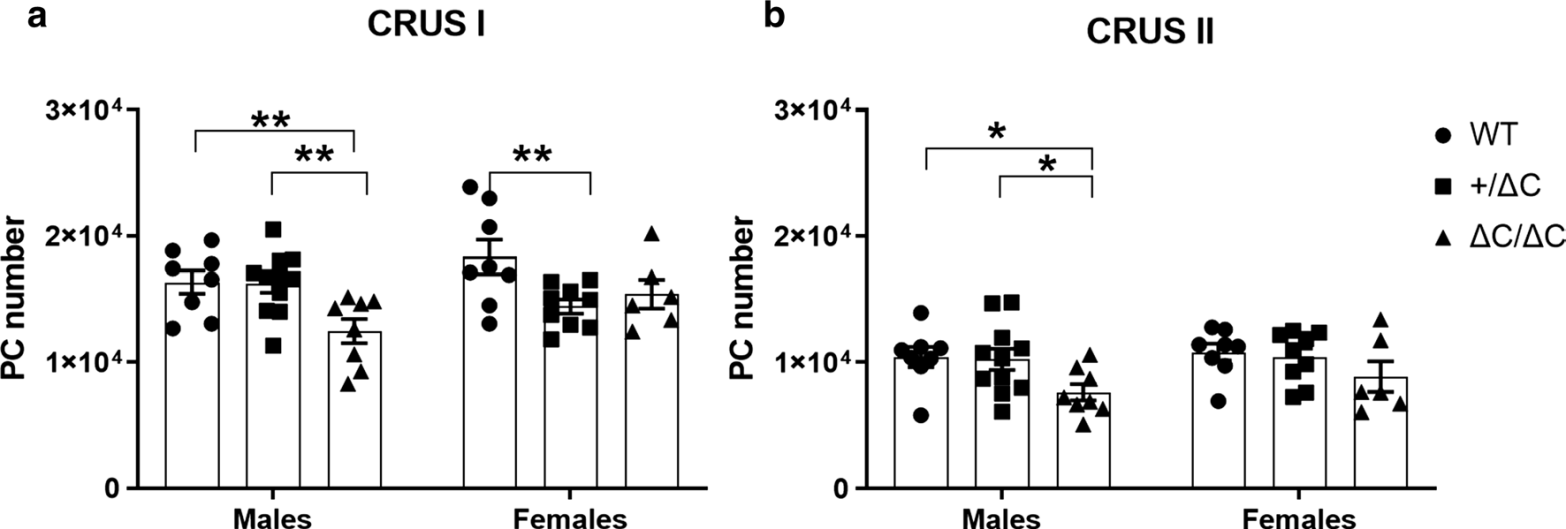
Purkinje cell (PC) number in *Shank3* mice. Stereological PC counts on coronal sections of the cerebellum in the Crus I (**a**) and the Crus II (**b**) sub-regions. Only male Shank3 ΔC/ΔC mice showed a decrease in the number of PC in the crus I and Crus II cerebellar sub-regions. Female + /ΔC mice showed a slight decrease in PC number in the Crus I subregion. All data are expressed as means ± SEM. Two-way ANOVA was performed (*p < 0.05, **p < 0.01, ***p < 0.001) WT males *n* = 8; Shank3+/ΔC males *n* = 11; Shank3 ΔC/ΔC males, *n* = 8; WT females *n* = 8; Shank3+/ΔC females *n* = 9; Shank3 ΔC/ΔC females, *n* = 6

Here, we report that the *Shank3* mutation resulted in a decreased number of PC in Crus I in both males and females (Fig. 6). Indeed, two-way ANOVA analysis showed a clear genotype (Two-way ANOVA, [F(2,44) = 5.92, *p* = 0.0053]) and sex effect (Two-way ANOVA genotype x sex interaction, [F(2,44) = 3.741, *p* = 0.032]). Fisher’s LSD post-hoc analysis further indicated that the Shank3 ΔC/ΔC male group had a lower number of PC than wild type in both the Crus I and Crus II cerebellar area (*p* = 0.0066 and *p* = 0.0206, respectively) (Fig. 6a, b). Females Shank3+/ΔC mice showed a mild but significant decrease in PC number in the Crus I subregion (Two-way ANOVA followed by Fisher’s LSD post-hoc analysis, [F(2,44) = 5.92, *p* = 0.005]) (Fig. 6a). In conclusion, the lower number of PC affects mainly Shank3 ΔC/ΔC male mice as also reported in the VPA and poly IC mouse models.

### Male Shank3 mice show decreased cerebellar mGluR5 levels

**Fig. 7:**
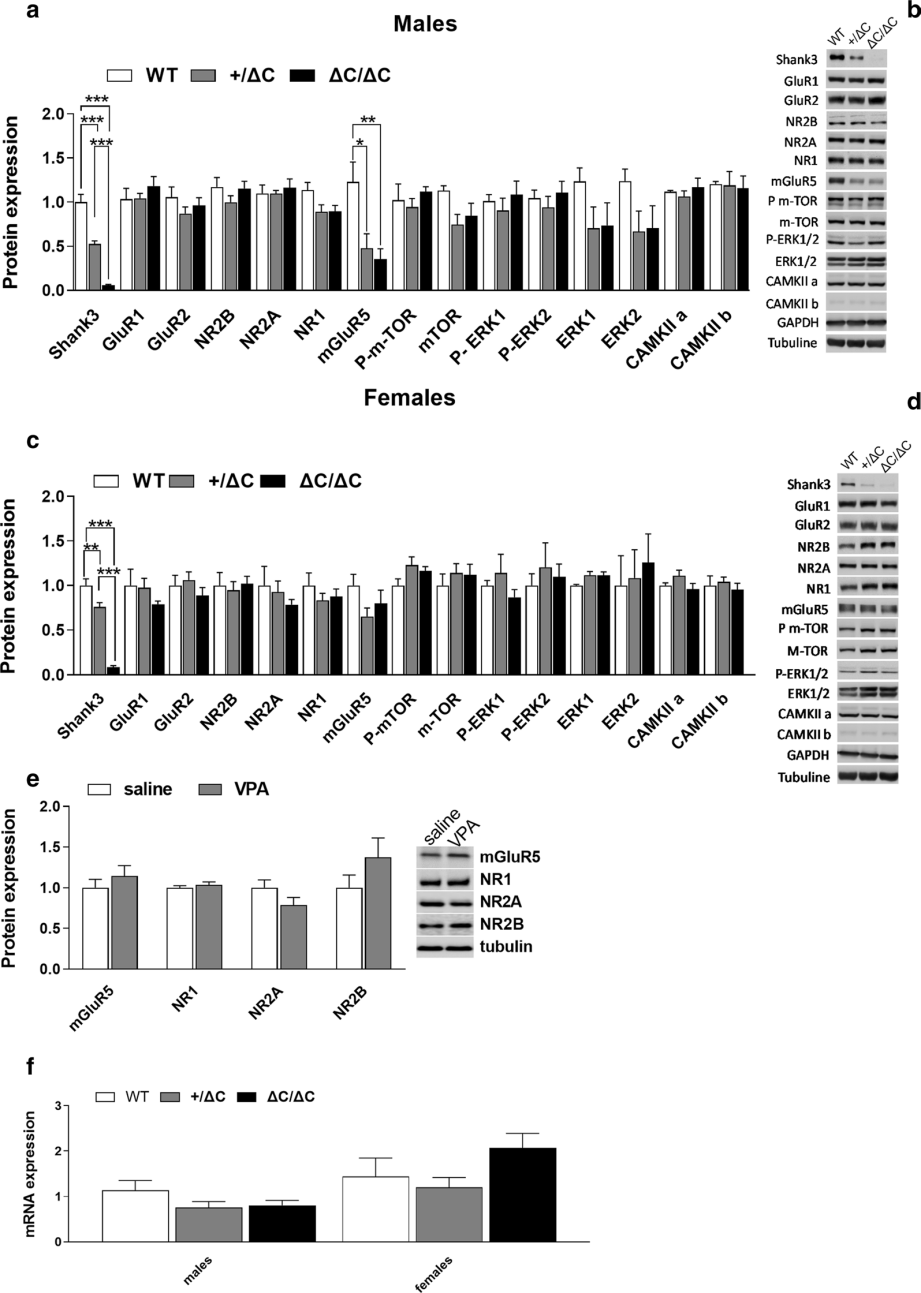
Brain levels in PSD proteins and mRNA in ASD mouse models. **a**–**c** Expression profile of Shank3 and associated postsynaptic scaffolding proteins in males and females *Shank3* mice within the cerebellum by immunoblot analysis. **b**–**d** Representative figures of the western blot. Note that only mGluR5 was significantly decreased in Shank3+/ΔC and ΔC/ΔC males compared to wild type. No decreases were present in Shank3 ΔC/ΔC and Shank3+/ΔC females. Experiments for western blots were repeated at least three times and statiscal analysis were performed with a one-way ANOVA followed by Tukey’smultiple analysis (*p < 0.05, **p < 0.01, ***p < 0.001) WT males *n* = 4; Shank3+/ΔC males *n* = 8; Shank3 ΔC/ΔC males, *n* = 8; WT females *n* = 5; Shank3+/ΔC females *n* = 8; Shank3 ΔC/ΔC females, *n* = 9. **e** No difference in the protein levels of several glutamate receptors was found in the male cerebellum of VPA ASD mouse model whatever the prenatal treatment (Saline, *n* = 5; VPA, *n* = 5, Student’s *t* test, *p* > 0.05). **f** No difference in the mGluR5 mRNA levels was found in the cerebellum whatever the genotype or sex (wild type males (*n* = 7), Shank3+/ΔC males (*n* = 8), Shank3 ΔC/ΔC males (*n* = 8), wild type females (*n* = 7), Shank3+/ΔC females (*n* = 8), Shank3 ΔC/ΔC females (*n* = 8), One-way ANOVA, *p* > 0.05). All data are expressed as means ± SEM

The *Shank3* C-terminal region (exon 21–22), which is mutated in these mice, contains binding sites for mGluR5 and plays a crucial role in the synaptic targeting and postsynaptic assembly of Shank3 scaffolding complex [43–45]. We thus explored the cerebellar levels of this receptor and associated scaffolding proteins, along with a set of other glutamate receptors and postsynaptic proteins (Fig. 7). Surprisingly, only mGluR5 levels were dramatically decreased and this was observed only in Shank3+/ΔC and Shank3 ΔC/ΔC males. Indeed, ANOVA analysis showed a genotype effect (One-way ANOVA, [F (2,16) = 6.368, p0.0092]) and Tukey’s multiple analysis revealed decreased levels of mGluR5 in males Shank3+/ΔC and Shank3 ΔC/ΔC mice compared to WT (*p* = 0.022 and *p* = 0.009, respectively). This decrease was dramatic in magnitude (down to − 71.32%) in male Shank3 ΔC/ΔC mice (Fig. 7a). In order to determine whether this decrease was specific to the *Shank3* mutation, we also evaluated levels of mGluR5, and NR1, NR2A and NR2B glutamate receptors’ subunits in a mouse model of prenatal VPA injection as described [28], and found no modifications in these levels (t(8) = 0.8569, *p* = 0.4164 for mGluR5, t(8) = 0.8427, *p* = 0.4239 for NR1, t(8) = 1.574, *p* = 0.1540 for NR2A, t(8) = 1.31, *p* = 0.2266 for NR2B). We further evaluated mGluR5 mRNA levels by RT-PCRq in order to determine whether the decrease in mGluR5 protein levels was due to transcriptional or translational regulations and found no alterations in the corresponding mRNA levels. Males: One-way ANOVA, [F(2,20) = 1.748, *p* = 0.1996]) and females: One-way ANOVA, [F(2,19) = 1.985, *p* = 0.1648]).

## Discussion

This study aimed at investigating several behavioral, cellular, and molecular outcomes related to ASD in Shank3+/ΔC and Shank3 ΔC/ΔC male and female mice with a mutation that is also observed in human conditions.

The *Shank3* gene encodes a master scaffolding protein in the postsynaptic density where it interacts with multiple key synaptic components, mainly implicating the glutamate receptor clusters, along with the cytoskeleton and signal transduction cascades [10, 26, 43, 44, 46]. The gene is located on chromosome 22q13.3 in humans and was first implicated in ASD in the 22q13.3 microdeletion syndrome, also known as Phelan-McDermid Syndrome [13]. Haploinsufficiency of the *Shank3* gene accounts for 0.5–2.0% of ASD and intellectual disability cases [16, 47]. Human genetic studies of ASD have found that the C-terminal region (exon 21) of *Shank3* harbors several mutations [16]. This region encodes binding sites for actin/Cortactin and mGluR5/Homer and plays a crucial role in the synaptic targeting and postsynaptic assembly of Shank3 scaffolding proteins [48]. A dozen different genetically alerted mice with various *Shank3* point mutations or deletions of a given exon have been constructed [17–19, 24, 26, 42, 49]. These mice show variable outcomes at the behavioral, cellular, and molecular levels.

We have set up our behavioral, cellular, and molecular analysis in a *Shank3* mouse model [17] based on our previous and recently reported findings in two different environmental mouse models of ASD [28, 29]. These models were obtained following prenatal injection of either VPA or poly IC and recapitulate several ASD symptoms, although at different degrees and magnitude, with a clear sexual dimorphism.

Here, we show that a *Shank3* mutation affects mostly male homozygote mice in several social and motor behavioral parameters. This is accompanied by a significant reduction in cerebellar PC and specific decreased levels of mGluR5 proteins. We first investigated social preference parameters, as they constitute one of the hallmarks of ASD [50]. We report that Shank3+/ΔC and Shank3 ΔC/ΔC males and females both have normal social preference and motivation but show impairments in social novelty preference. Of interest is the fact that ASD patients are reported to avoid unfamiliar social partners and display diminished interest in novelty [50]. Indeed, ASD patients tend to avoid social contacts with a new individual compared to someone familiar [51].

Self-grooming is a core behavior for mice that spend about 40% of their waking time performing it. It is aimed to maintain physiological stasis and comfort and thus tends to increase under stressful situations and in pathological conditions implicating motor brain centers as reported in clinical and animal models of ASD [33]. Here, we replicated findings in these animal models showing increased grooming, reflective of stereotyped repeated behavior. We further show that this increased grooming affects both Shank3 ΔC/ΔC males and females.

In the initial paper using this line of mice [17], authors have investigated a wide range of behavioral parameters including motor-coordination deficits, grooming and novelty avoidance, but only in homozygote mice. Of interest is the observation that homozygote mice showed social preference in the phase 2 of the 3-CT but no preference for social novelty (phase 3), a finding that we replicated here both in males and females. In the report by Duffney et al., (2015), Shank3+/ΔC did show social preference in phase 2 trial but that was of a lesser magnitude than in wild type animals [18]. Qin et al. [19] used the 3-CT with only two phases and showed no social preference in Shank3+/ΔC mice, mixing measurements of ‘investigating behavior” and time spent in the area surrounding the cup with the social cue. Using a similar mutation leading to a premature stop codon in exon 21 of *Shank3*, Speed et al. [24] found no social interaction deficits in both heterozygote or homozygote mouse mutants. Also in this paper, homozygote mice, but not heterozygotes, showed deficits in hippocampus-dependent spatial learning, impaired motor coordination, altered response to novelty, and sensory processing deficits. Jaramillo et al. [22] used a different approach to generate Shank3 mutant mice with a loss of the two higher molecular weight isoforms by disrupting the PDZ domain with a transcriptional stop cassette prior to exon 13. Homozygote mice with this mutation, but not heterozygotes, displayed a preference for the social target over the inanimate object (phase 2), a finding that we replicated here. In the phase 3 of the 3-CT, neither heterozygote nor homozygote mice displayed preference to social novelty as also we show here for homozygote mice. In addition, interaction time and approach were altered only in homozygote animals, in line with our findings of altered social behavior only in homozygotes. In the grooming behavior, both heterozygotes and homozygotes displayed increased time spent grooming compared to wild type, but only homozygotes showed increased grooming bouts, a finding that we also replicate in the *Shank3* mouse models that we used. Although apparently conflicting, collectively these results show alterations in grooming and social behavior in *Shank3* mutant mice, which are often present only in homozygotes or at least with a much higher degree than in heterozygote mice.

Several reports in ASD described impairments in visio-motor and manual dexterity tasks, limb coordination during tasks requiring balance, agility, and speed, as well as in gait and ataxia [52]. Furthermore, motor impairments may be amongst the earliest signs of some forms of ASD and their assessment might help the early and quantitative diagnosis of the pathology and the identification of dysfunctional brain regions and circuits in ASD [53]. Here, we found only mild motor coordination impairments in this mouse model. These impairments were revealed only when the task required significant challenge (a very narrow beam with a grid mech above it). This is in line with our findings with a Poly IC environmental model where no deficiencies were observed in this task [29].

One of the most robust and consistent findings in our previous studies and here are anomalies in gait. Early studies in ASD patients have revealed irregular gait [54]. Quantitative measures indicated increased variability and irregularity in gait suggestive of cerebellar, rather than basal ganglia, involvement. Later studies reported increased missteps, increased step width, and higher ataxia ratios similar to what is found in patients with cerebellar lesions [55]. In fact, several spatiotemporal gait parameters of children with ASD were similar to those of patients with cerebellar ataxia [56]. Of interest is the fact that abnormal gait patterns seem to be correlated with the severity of social impairments in both humans [57] and ASD animal models [28]. Here, we have used an automated rodent quantitative gait analysis system with optical touch sensors, which records and tracks rodents’ footprints as they move freely in a walkway. This allowed qualitative and quantitative readout of footprint pattern and contacts, highlighting gait abnormalities. As nearly all gait parameters are correlated, a collection of these parameters is needed to determine the potential alterations’ nature. Gait is made up of strides that comprise a stance phase with the foot in contact with the ground and a swing phase with the foot off the ground. Fundamental descriptors of gait can be divided into two main sections: (1) spatial patterns that include stride length, step length, and step width and (2) temporal parameters including duty factor (limb stance time divided by stride time) and limb phase (the time between forelimb and hindlimb foot-strike on the same side divided by the stride time). We report here that several temporal parameters were altered and that these alterations were mainly found in Shank3 ΔC/ΔC mice and to a wider extend in males. Interestingly, spatial patterns did not seem to be altered by the mutation (stride length did not differ between groups), indicating no major anatomical alterations in these mice. However, all temporal parameters measured were reduced in Shank3 ΔC/ΔC mice (stance time, swing time, stride frequency, and duty factor). Along with these temporal deficiencies, there were also alterations of the way paws are applied to the surface in terms of surface contact and strength, and that were also reduced mainly in both males and females Shank3 ΔC/ΔC. Also of interest is the fact that more gait parameters were affected in males than in females. The temporal and surface alterations observed are probably not due to change in velocity as only females, but not males, Shank3 ΔC/ΔC mice showed a mild increase (less than 20%) in velocity; in addition, there was no change in the regularity of gait nor the stride length. Altogether, these gait elements argue against an anatomical alteration and a primary motor disorder implicating the basal ganglia. In contrast, they seem to point to a cerebellar dysfunction as they mainly affect movement coordination, a cerebellar function, which is in line with clinical findings (see for instance reference [56]).

Gait disturbances, and more generally behavioral abnormalities in neurodevelopmental disorders including ASD, are a consequence of underlying alterations in circuit maturation and function. Post-mortem and brain imaging studies have consistently identified the cerebellum as one of the most abnormal brain regions associated with ASD with a specific reduction of cerebellar PC [40, 41]. Here we show a decreased number of PC within the hemispheric part of lobule VII, within the in Crus I and Crus II subregions and only in Shank3 ΔC/ΔC males. Females showed only a mild decrease in PC number in Shank3+/ΔC. This is in line with VPA and Poly IC models showing also decreases of PC in these brain areas and that were more pronounced in males than in females. Cerebellar defects seem to be widespread in ASD mouse models. Using five different ASD mouse models, including the one used in this study, Kloth et al. [49] have shown major cerebellar associative sensory learning deficits with the eyeblink conditioning task, a task that relies on cerebellar plasticity. Further on, mice with a Tsc1 mutation specifically in cerebellar cells PC showed ASD-related altered behavior that was rescued with rapamycin treatment targeting mTOR signaling further pointing to these cells as key players in ASD [42].

In this line, we investigated the levels of dozens of cerebellar proteins implicated in the cerebellar glutamatergic transmission and signal transduction. In accordance with previous findings in cortical or hippocampal areas, we did not detect any difference in the cerebellar expression level of several glutamatergic related proteins [18, 19, 24]. We further extend these findings and show here a dramatic decrease in the levels of cerebellar mGluR5 protein, and only in male Shank3 ΔC/ΔC, pointing to that receptor as the major target of Shank3 and perhaps even the major component underlying the observed behavioral deficits linked to this mutation. These decreased protein levels were probably due to post-translational regulation as we did not detect any alterations in the corresponding mRNA levels. Of interest is also the fact that such a decrease was not observed in the VPA environmental ASD mouse model. Shank3 is essential to mediating mGluR5 signaling by recruiting Homer1b/c to the postsynaptic density [17, 18, 44]. Pharmacological enhancement of mGluR5 activation in *Shank3* knock-out mice, through the administering 3-Cyano-*N*-(1,3-diphenyl-1H-pyrazol-5-yl)benzamide, ameliorated functional and behavioral defects [58], suggesting that pharmaceutical treatments that increase mGluR5 activity may represent an interesting approach for treating ASD patients with a *Shank3* mutations [18, 59, 60]. In this line, restoration of Shank3 expression in adult using a *Shank3* conditional knock-in procedure in mice, rescued several ASD phenotypes through improvements in synaptic protein composition, spine density, and neural function [27]. This is of interest as it further emphasis the major role of Shank3 in postsynaptic function and indicates that this neurodevelopmental pathology may in fact be manageable at adult age in patients with *Shank3* mutations.

*Shank3* mutations in ASD patients are either point mutations in one copy of the *Shank3* gene or haploinsufficiencies where a single functional *Shank3* allele is insufficient to maintain a normal behavior [14, 61]. Thus, one would have expected that Shank3+/ΔC mice would show clear behavioral alterations relevant to ASD. In our hands however, Shank3+/ΔC mice showed deficiencies in the social novelty recognition task and did not show other behavioral or cellular dysfunction, despite over a 50% decrease in mGluR5 expression. This is in line with previous publications with this or similar mouse model [17, 24] but at odds with other previous reports exploring behavioral and cellular consequences only in males [18, 19].

Several studies utilizing *Shank3* mutations or deletions do not report measurable changes in animal behavior. For instance, exons 4–9 of *Shank3* (JAX 017890) show only mild ASD-related phenotype and have normal sociability and social novelty in the 3-chamber test [61]. Mutations targeting exons 4–7 only yielded mild behavioral phenotypes, while targeting exons 13–16 (JAX 017688) showed profound phenotypes such as impaired sociability and preference for social novelty, as well as reduced pair interaction, and profound self-grooming leading to skin lesions [62]. Targeting exon 11 induced prominent self-grooming and skin lesions [63], while targeting exon 21 of *Shank3* (JAX 018398) results in normal sociability but impaired social novelty in the 3-chamber test [17], as observed in our study here.

## Limitations

The construct validity of several, but not all, of the *Shank3* mouse lines is strong as they tend to reproduce human mutations. However, several of these mouse models may have limited face validity, given that they sometimes fail to reproduce behavioral and cellular symptoms reported in ASD patients. Another element to take into consideration is the fact that each of these mutants targeted only a subset of *Shank3*′s coding exons. Consequently mice only lacked a subset of *Shank3* protein isoform, which may have differential function across brain regions, developmental time periods, or cell types [64]. Another limitation is the possibility that some *Shank3* mutations lead only to subtle or limited forms and symptoms in the autism spectrum and that are difficult to detect in mice (such as intellectual disabilities).

## Conclusion

Several major conclusions can be drawn from our study and several previous related findings: (1) the need to include females and heterozygote mice in separate groups in ASD animal models. (2) Even though *Shank3* mutations have strong construct validity when produced in a way to mimic the genetic deletions and mutations observed in humans, there is a need to better evaluate Shank3 mouse models’ face validity. For instance, in our hands and in previous reports [17, 24], Shank3+/ΔC did not show major ASD-related behavioral deficits, contrary to the haploinsufficiency *Shank3* deletions in humans. (3) Our findings, along with our previous reports in environmental mouse models of ASD, clearly point to gait disturbances as being key behavioral features in ASD, whatever the disease’s etiology, with symptoms that are in line with clinical findings. This is of interest as gait investigations can be implemented early during the diagnosis procedures when ASD is suspected; they are also quantitative in an objective and reproducible manner. As such, gait needs to be considered as a credible new diagnostic criterion in ASD.

## Data Availability

Most of the data generated and analyzed during this study are included in this published article. All datasets used and/or analyzed during the current study are available from the corresponding author on reasonable request.

## Abbreviations

SHANK: SH3 and multiple ankyrin repeat domains
ASD: Autism Spectrum Disorders
PC: Purkinje cells
ANOVA: Analysis of variance
mGluR5: Metabotropic glutamate receptor 5
NRXN: Neurexin
NLGN: Neuroligin
TSC1/2: Tuberous sclerosis 1/2
FMR1: Fragile Mental Retardation 1
VPA: Valproic acid
MIA: Maternal immune activation
3-CT: Three-chambers test
PFA: Paraformaldehyde
SDS: Sodium dodecyl sulfate
TBST: Tris Buffer Solution Tween
P-mTOR: Phosphorylated mammalian target of rapamycin
mTOR: Mammalian target of rapamycin
ERK1/2: Extracellular signal regulated kinase
NR2A: *N*-Methyl-d-aspartate receptor subunit 2A
NR2B: *N*-Methyl-d-aspartate receptor subunit 2B
GluR1: α-Amino-3-hydroxy-5-methyl-4-isoxazolepropionic acid receptor subunit 1
GAPDH: D-Glycéraldéhyde-3-phosphate déshydrogénase
NR1: *N*-Methyl-d-aspartate receptor subunit 1
GluR2: α-Amino-3-hydroxy-5-methyl-4-isoxazolepropionic acid receptor subunit 2
